# Human immunodeficiency virus care cascade among sub-populations in Rakai, Uganda: an observational study

**DOI:** 10.7448/IAS.20.1.21590

**Published:** 2017-06-05

**Authors:** Veena G. Billioux, Larry W. Chang, Steven J. Reynolds, Gertrude Nakigozi, Joseph Ssekasanvu, Mary K. Grabowski, Robert Ssekubugu, Fred Nalugoda, Godfrey Kigozi, Joseph Kagaayi, David Serwadda, Ronald H. Gray, Maria J. Wawer

**Affiliations:** ^a^ Department of Epidemiology, Johns Hopkins Bloomberg School of Public Health, Baltimore, MD, USA; ^b^ Department of Rakai Community Cohort Study, Rakai Health Sciences Program, Kalisizo, Uganda; ^c^ Division of Infectious Diseases, Department of Medicine, Johns Hopkins School of Medicine, Baltimore, MD, USA; ^d^ Laboratory of Immunoregulation, Division of Intramural Research, National Institute of Allergy and Infectious Diseases, National Institutes of Health, Bethesda, MD, USA; ^e^ Department of Disease Control & Environmental Health, School of Public Health, Makerere University, Kampala, Uganda

**Keywords:** HIV, antiretroviral, HIV care cascade, Rakai, Uganda

## Abstract

**Introduction**: To assess progress towards the UNAIDS 90–90–90 initiative targets, we examined the HIV care cascade in the population-based Rakai Community Cohort Study (RCCS) in rural Uganda and examined differences between sub-groups.

**Methods**: Self-reports and clinical records were used to assess the proportion achieving each stage in the cascade. Statistical inference based on a χ^2^ test for categorical variables and modified Poisson regression were used to estimate prevalence risk ratios (PRRs) and 95% confidence intervals (CI) for enrolment into care and initiating antiretroviral therapy (ART).

**Results**: From September 2013 through December 2015, 3,666 HIV-positive participants were identified in the RCCS. As of December 2015, 98% had received HIV Counseling and Testing (HCT), 74% were enrolled in HIV care, and 63% had initiated ART of whom 92% were virally suppressed after 12 months on ART. Engagement in care was lower among men than women (enrolment in care: adjPRR 0.84, 95% CI 0.77–0.91; ART initiation: adjPRR 0.75, 95% CI 0.69–0.82), persons aged 15–24 compared to those aged 30–39 (enrolment: adjPRR 0.72, 95% CI 0.63–0.82; ART: adjPRR 0.69, 95%CI 0.60–0.80), unmarried persons (enrolment: adjPRR 0.84, 95% CI 0.71–0.99; ART adjPRR 0.80, 95% CI 0.66–0.95), and new in-migrants (enrolment: adjPRR 0.75, 95% CI 0.67–0.83; ART: adjPRR 0.76, 95% CI 0.67–0.85). This cohort achieved 98–65–92 towards the UNAIDS “90–90–90” targets with an estimated 58% of the entire HIV-positive RCCS population virally suppressed.

**Conclusions**: This cohort achieved over 90% in both HCT and viral suppression among ART users, but only 65% in initiating ART, likely due to both an ART eligibility criterion of <500 CD4 cells/mL and suboptimal entry into care among men, younger individuals, and in-migrants. Interventions are needed to promote enrolment in HIV care, particular for hard-to-reach sub-populations.

## Introduction

Through viral suppression, effective antiretroviral therapy (ART) prevents progression to AIDS [1–3] and death [4,5], and substantially reduces HIV transmission [6,7], thus helping to curtail the HIV epidemic [8,9]. Given these benefits of ART, the Joint United Nations Programme on HIV/AIDS (UNAIDS) has set a “90–90–90” target by 2020 to diagnose and counsel 90% of all HIV-positive individuals, provide ART for 90% of those diagnosed as HIV positive, and achieve sustained viral suppression for 90% of those treated. This translates to 73% of all HIV-positive individuals being virally suppressed [10].

Reaching the UNAIDS targets requires early diagnosis, effective treatment, and maintaining patients in care [11–13]. However, there is growing evidence that, even among HIV-positive individuals who know their status, substantial proportions do not enrol into HIV care and treatment programs. Delays in diagnosis and entry into care lead to late presentation for ART, with increased risks of HIV-related morbidity and mortality and continued viral transmission [14]. In addition, patients who enrol in HIV care are sometimes non-adherent and do not achieve viral suppression, or are not effectively retained in care [15].

Gardner and colleagues described levels of engagement in HIV care, ranging from “unaware of HIV infection” to “fully engaged in HIV care with suppressed viral load” [16], i.e., the HIV “care cascade” framework. The framework provides a population-based approach to programme monitoring and highlights opportunities for intervention. The Rakai Community Cohort Study (RCCS), a large and long-standing population-based cohort, offers a unique opportunity to study select stages of the HIV care cascade in a rural East African population. Most HIV care programmes are clinic based and do not have population-based data with which to determine the proportion of HIV-positive individuals or selected subgroups who do or do not access care. We assessed the spectrum of engagement in care and examined differences between sub-groups of HIV-positive individuals enrolled in the Rakai Community Cohort Study in Rakai Uganda.

## Methods

### Study population

The RCCS, ongoing since 1994, is a longitudinal population cohort of approximately 17,000 persons aged 15–49 years conducted by the Rakai Health Sciences Program (RHSP). The RHSP is also a US President’s Emergency Plan for AIDS Relief (PEPFAR) implementer, providing HIV counseling and testing (HCT), pre-ART care, and ART. All HIV-positive persons identified via the RCCS are referred for care and treatment. Beginning in September 2013, RHSP transitioned from directly delivering care in 19 government health facilities to Ministry of Health (MOH)-led delivery of services (District Lead Programming – DLP) with RHSP assistance. The population for this study included HIV-positive residents of the region enrolled in the RCCS between September 2013 and December 2015; corresponding to when Ministry of Health-led delivery of services began in the study region.

The RCCS includes 41 agrarian, trading, and fishing communities in the region of Rakai District, south-central Uganda. RCCS communities are representative of rural Uganda (HIV prevalence is ~14% in trading communities, 12% in agrarian communities and ~42% in high-risk fishing communities [17]. All RCCS communities are within an hour’s walk of a clinic, the majority being within half hour by foot.

At approximately 18-month intervals, structured confidential RCCS interviews are conducted in Luganda, the local language, by trained same-sex interviewers, in order to collect information on sociodemographic characteristics, health (including the use of HIV care and ART), and sexual risk behaviours. Prior to the interview, pretest counselling and HIV testing is offered free of charge using a validated three rapid test algorithm [18,19], and participant who consent to receive their HIV results receive post-test counselling by on-site counsellors. RHSP staff also collected detailed data to link clinic patients to the RCCS survey participants.

HIV care is provided by MOH personnel, with supervisory and monitoring support from the RHSP. In the clinics, pre-ART HIV care consists of cotrimoxazole for opportunistic infection prophylaxis, bed nets for malaria prevention, and clean water vessels with hypochlorite to prevent diarrhoea, positive prevention education, reproductive health services and treatment of sexually transmitted infections. Six monthly CD4+ cell count monitoring is used to assess ART eligibility. Since January 2014 the criteria for ART initiation were raised to a CD4 cell count ≤500 cells/mm^3^ (from ≤350 cells/mm^3^) for the general population, and test and treat for most-at-risk populations in fishing communities [20]. First-line ART consists of standard three-drug regimens approved by the Uganda MOH. HIV-positive individuals on ART are monitored clinically and via six monthly CD4+ cell counts and HIV viral load assays. However, the viral load testing is conducted at a central national laboratory, and there have been delays in the return of results; thus, viral load measurements 12 months after ART initiation were only available for a fraction of ART patients (20%, 366/1850).

The study was reviewed and approved by the Uganda Virus Research Institute Research and Ethics Committee, the Uganda National Council on Science and Technology, the Johns Hopkins University School of Medicine Institutional Review Board, and the Western Institutional Review Board, Olympia, WA. Study participants provided written informed consent at each RCCS visit; the consent included agreement to link participants’ RCCS survey results to their clinical data.

### Data sources

For this analysis, we linked two longitudinal data sources: the RCCS survey and surveillance data maintained by RHSP and the electronic RHSP clinical data system which uses the Open Medical Record System (OpenMRS) an open-source electronic health record [21]. The RHSP clinical data system is derived from the local Ministry of Health clinic-based HIV treatment and care information system, which contains data for all patients enrolled in either pre-ART or ART in each of the 19 clinics within the Rakai district. We linked the OpenMRS data at the individual level to the data in the RCCS study system by using the laboratory identification number utilized in RHSP supported clinics. Together, these data provide information on HIV prevalence in the community, and on the HIV care cascade. The RCCS ascertains the proportion of HIV-positive individuals who accept HCT from RCCS counsellors and also collects self-reported data on receipt of HCT from the RCCS or other providers, engagement in pre-ART care and receipt of cotrimoxazole, and use of ART. HIV care status was also assessed from clinic-based patient records. Clinic data included date of visit; cotrimoxazole and ART dispensed; blood samples for CD4+ cell counts and HIV viral load testing; patient health status and laboratory results when available. Mortality and outmigration prior to December 2015 were ascertained using both the clinical and RCCS study records and individuals who died or out-migrated were removed from the population at risk denominator.

### HIV care cascade outcomes

Four stages of the HIV care cascade were included in our framework:

- Awareness of positive HIV status was defined as having received HCT test results through the RCCS counsellors and/or self-reported receipt of HCT at a time point after their first positive test identified through the RCCS.
- Enrolment in HIV care was defined as completing at least 1 clinic visit and/or self-reported use of cotrimoxazole or ART.
- ART status was defined by having a clinically confirmed ART initiation date and/or self-reported use of ART.
- Viral suppression was defined as a viral load ≤1000 copies/mL 12 months after initiation of ART per WHO recommendations [22]. Since viral load testing was not available for participants who self-reported ART from other HIV care providers, the proportion of participants who were virally suppressed was estimated among ART recipients with a viral load measurement 12 months after ART initiation based on RHSP and MOH clinic records.

### Statistical analysis

Participants were categorized into the cascade categories described above. The proportion of HIV-positive persons achieving each stage in the cascade was calculated, and statistical inference was based on a χ^2^ test for categorical variables. We also used modified Poisson regression to estimate prevalence risk ratios (PRRs) and 95% confidence intervals (95%CIs) of enrolment into care and initiation of ART. Covariates associated with enrolment into care and initiation of ART in the bivariate analyses with *p* values <0.05 and potential confounders identified in the literature were included in the multivariable models. In a sensitivity analysis of viral suppression, we used inverse probability weighting to account for potential selection bias associated with having a viral load measurement 12 months after ART initiation. Inverse probability weights were constructed based on established methods [23] using a logistic regression model and data on the age, education level, occupation, socioeconomic status, community type, and migration status of participants with and without a viral load measurement 12 months after ART initiation. Weighted PRRs were estimated using Poisson regression assuming independence between individual participant observations and conditional on observed covariates. Migrants were identified through the RCCS community census and defined as persons who moved from another community regardless of distance travelled. For classification of socioeconomic status, we used a household wealth index, based on the building materials of the respondent’s home [24]. All statistical analyses were performed in the R statistical software (V3.2.5), and the inverse probability weighted analysis was done using the survey package.

## Results

From September 2013 through December 2015, a total of 3,666 HIV-positive participants were identified in the Rakai Community Cohort (Table 1). In total, 63% (2308/3666) of HIV-positive participants were female. The median age of all HIV-positive participants was 33 years (Interquartile range, IQR, 27–38). 59% (2166/3666) were currently married, 88% (3207/3666) were Christian, and 92% (3367/3666) had at least some primary education.

**Table 1.** Characteristics of 3666 HIV-positive persons enrolled in the Rakai Community Cohort Study, December 2015

Characteristic	N	(%)
**Total**	3666	(100.0)
**Female**	2308	(63.0)
**Male**	1358	(37.0)
**Age, Years^a^**	33	(27–38)
15–24	542	(14.8)
25–29	776	(21.2)
30–39	1611	(43.9)
40+	737	(20.1)
**Marital status**		
Married	2166	(59.1)
Never married	282	(7.7)
Previously married	1218	(33.2)
**Religion**		
Christian	3207	(87.5)
Muslim	429	(11.7)
Other	30	(0.8)
**Education**		
No education	101	(2.8)
Some primary	3367	(91.8)
Post-primary	198	(5.4)
**Occupation**		
Agriculture	638	(17.4)
Home/casual/other	1089	(29.7)
Shop/skilled worker	297	(8.1)
Bar/waitress/sex worker	392	(10.7)
Fisherman	551	(15.0)
Trade/truck or motorcycle driver	699	(19.1)
**Wealth index**		
High	1631	(44.5)
Middle	723	(19.7)
Low	1312	(35.8)
**Long-term resident**	2888	(78.8)
**In-migrant**	778	(21.2)
**Community type**		
Agrarian	1048	(28.6)
Fishing	1743	(47.5)
Trading	875	(23.9)

^a^Median (IQR).

**Figure 1.**
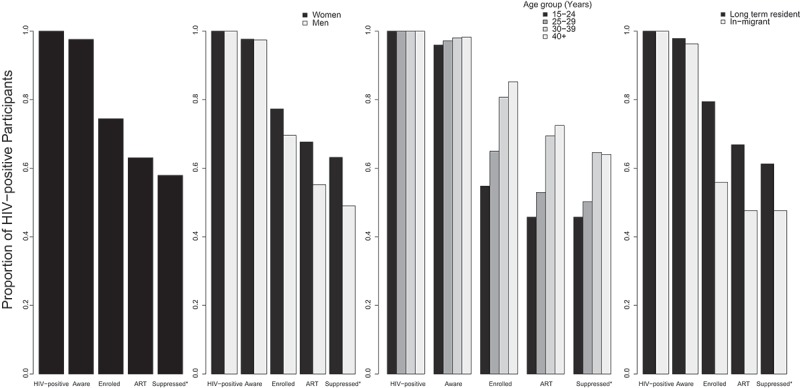
Proportion of RCCS participants in December 2015 in each of the HIV care cascade stages by selected characteristics, Rakai, Uganda. *Imputed based on the proportion suppressed with a viral load measurement 12 months after initiating ART.

Figure 1 and Table 2 show the HIV care cascade by participant characteristics. In total, 98% (3577/3666) of all HIV-positive participants were aware of their status; 92% (3386/3666) had consented to and received HIV test counselling, and 5% (191/3666) self-reported receiving their HIV test results and were thus aware of their status. 74% (2729/3666) were enrolled in HIV care, and 63% (2312/3666) had initiated ART. We found that 76% (2729/3577) of those who knew their results were in care; the higher rate of those in care compared to those on ART was due in part to the CD4 initiation criteria during this period (CD4 < 500 cells/mm^3^). Among 1288 persons who had CD4 measurements available, 96% (1143/1186) of those found to be eligible, given the criteria at the time, were on ART. Among 366 persons who had a viral load measurement 12 months after initiating ART, 92% (336) had a suppressed viral load. Extrapolating to the whole population of HIV-positive RCCS participants, we estimate that 58% (2124/3666) of the overall population of HIV-positive participants were virally suppressed. The inverse probability weighted estimate of viral suppression was 57%.

**Table 2.** Proportion of Rakai Community Cohort Study population engaged in each of the HIV care cascade stages, Rakai, Uganda

	Aware of HIV status			Enrolled in care			Initiated ART			Virally suppressed		
Characteristic	*n*/*N*	%	*p*-Value*	n/N	%	*p*-Value*	n/N	%	*p*-Value*	n/N	%	*p*-Value*
**Total**	3577/3666	97.6		2729/3666	74.3		2312/3666	62.9		2124/3666	57.7	
**Female**	2254/2308	97.7		1784/2308	77.3		1562/2308	67.7		1458/2308	63.2	
**Male**	1323/1358	97.4	0.658	945/1358	69.6	**0.000**	750/1358	55.2	**0.000**	666/1358	49.0	**0.000**
**Age, Yrs.**												
15–24	520/542	95.9		297/542	54.8		248/542	45.8		248/542	45.8	
25–29	754/776	97.2		504/776	64.9		411/776	53.0		390/776	50.3	
30–39	1579/1611	98.0		1300/1611	80.7		1119/1611	69.5		1041/1611	64.6	
40+	724/737	98.2	**0.033**	628/737	85.2	**0.000**	534/737	72.5	**0.000**	472/737	64.0	**0.000**
**Marital status**												
Married	2115/2166	97.6		1630/2166	75.3		1386/2166	64.0		1265/2166	58.4	
Never married	270/282	95.7		166/282	58.9		133/282	47.2		124/282	44.0	
Previously married	1192/1218	97.9	0.128	933/1218	76.6	**0.000**	793/1218	65.1	**0.000**	734/1218	60.3	**0.000**
**Religion**												
Christian	3136/3207	97.8		2394/3207	74.6		2024/3207	63.1		1843/3207	57.5	
Muslim	412/429	96.0		314/429	73.2		269/429	62.7		262/429	61.1	
Other	29/30	96.7	0.074	21/30	70.0	0.661	19/30	63.3	0.980	19/30	63.3	0.311
**Occupation**												
Agriculture	624/638	97.8		481/638	75.4		419/638	65.7		372/638	58.3	
Home/casual/other	1066/1089	97.9		846/1089	77.7		731/1089	67.1		672/1089	61.7	
Shop/skilled worker	282/297	94.9		201/297	67.7		175/297	58.9		156/297	52.5	
Bar/waitress/sex worker	381/392	97.2		302/392	77.0		254/392	64.8		238/392	60.7	
Fisherman	540/551	98.0		382/551	69.3		295/551	53.5		268/551	48.6	
Trade/truck or motorcycle driver	684/699	97.9	0.127	517/699	74.0	**0.001**	438/699	62.7	**0.000**	422/699	60.4	**0.000**
**Wealth index**												
High	1584/1631	97.1		1211/1631	74.2		1041/1631	63.8		953/1631	58.4	
Middle	705/723	97.5		508/723	70.3		431/723	59.6		389/723	53.8	
Low	1288/1312	98.2	0.188	1010/1312	77.0	**0.002**	840/1312	64.0	0.099	786/1312	59.9	**0.029**
**Long-term resident**	2826/2888	97.9		2294/2888	79.4		1931/2888	66.9		1770/2888	61.3	
**In-migrant**	749/778	96.3	**0.019**	435/778	55.9	**0.000**	371/778	47.7	**0.000**	371/778	47.7	**0.000**
**Community type**												
Agrarian	1035/1063	97.4		793/1063	74.6		680/1063	64.0		627/1063	59.0	
Fishing	1688/1721	98.1		1298/1721	75.4		1076/1721	62.5		1048/1721	60.9	
Trading	854/882	96.8	0.110	638/882	72.3	0.241	556/882	63.0	0.762	475/882	53.9	**0.002**

*Fisher’s Chi-squared *p*-value. Bold values indicate *p* < 0.05.

There was no significant difference by sex in the receipt of HIV test results (*p* = 0.658), but there were significantly lower proportions of males than females in every subsequent stage of the HIV care cascade (*p* < 0.000, Table 2). Individuals aged 15–24 were less likely to be aware of their HIV status (*p* = 0.033), to be enrolled in care (*p* < 0.000), on ART (*p* < 0.000) and have a suppressed viral load (*p* < 0.000), compared to HIV-positive persons aged 30–39 years. In addition, persons who had in-migrated were less likely to be aware of their HIV status (*p* = 0.019), to be enrolled in care (*p* < 0.000), on ART (*p* < 0.000) and less likely to have a suppressed viral load (*p* < 0.000) than long-term residents. We found that the majority (85% [2312/2729]) of those enrolled in care had initiated ART; initiation was 83% [1076/1298] in fishing communities receiving test and treat, which was similar to 86% [680/793] in Agrarian communities, and 87% [556/638] in trading communities in which ART was initiated at a CD4 count <500 cells/mm^3^. Despite the fact that fishing community populations were offered ART at the time of diagnosis, we found the same disparities for entry into care and ART initiation by age, sex, marital status, and migration status (Supplementary Table 1).

Table 3 shows the unadjusted and adjusted PRR of enrolment into care for all participants. Men were less likely to be enrolled in care compared with women (adjPRR 0.84, 95% CI 0.77–0.91). Enrolment into care by HIV-positive participants aged 15–24 was 28% lower than among older individuals aged 30–39 (adjPRR 0.72, 95% CI 0.63–0.82). Never married HIV-positive participants, were less likely to be enrolled in care compared with married individuals (adjPRR 0.84, 95% CI 0.71–0.99), and in-migrants had 25% lower enrolment in care than long-term residents (adjPRR 0.75, 95% CI 0.67–0.83). There was no difference in enrolment in care in trading communities (adjPRR 0.98, 95% CI 0.88–1.09) or in fishing communities receiving test and treat (adjPRR 1.08, 95% CI 0.98–1.18) compared to agrarian communities.

**Table 3.** Unadjusted and adjusted prevalence risk ratio (PRR) for enrolment into care, Rakai, Uganda

	Unadjusted			Adjusted		
Characteristic	PRR	95% CI	*p*-Value	PRR^a^	95% CI	*p*-Value
Female	ref			ref		
Male	0.90	(0.83–0.97)	**0.009**	0.84	(0.77–0.91)	**0.000**
**Age, Years**						
15–24	0.68	(0.60–0.77)	**0.000**	0.72	(0.63–0.82)	**0.000**
25–29	0.80	(0.73–0.89)	**0.000**	0.83	(0.75–0.92)	**0.001**
30–39	ref			ref		
40+	1.06	(0.96–1.16)	0.263	1.05	(0.96–1.16)	0.290
**Marital status**						
Married	ref			ref		
Never married	0.78	(0.66–0.91)	**0.003**	0.84	(0.71–0.99)	**0.038**
Previously married	1.02	(0.94–1.10)	0.666	0.96	(0.89–1.05)	0.384
**Religion**						
Christian	ref			ref		
Muslim	0.98	(0.87–1.10)	0.743	0.98	(0.87–1.11)	0.798
Other	0.94	(0.59–1.40)	0.769	0.96	(0.60–1.43)	0.843
**Occupation**						
Agriculture	ref			ref		
Home/casual/other	1.03	(0.92–1.15)	0.600	1.03	(0.92–1.16)	0.588
Shop/skilled worker	0.90	(0.76–1.06)	0.199	0.95	(0.81–1.12)	0.575
Bar/waitress/sex worker	1.02	(0.88–1.18)	0.768	1.04	(0.90–1.20)	0.619
Fisherman	0.92	(0.80–1.05)	0.221	1.03	(0.88–1.20)	0.749
Trade/truck or motorcycle driver	0.98	(0.87–1.11)	0.763	1.01	(0.89–1.14)	0.917
**Wealth index**						
High	ref			ref		
Middle	0.95	(0.85–1.05)	0.297	0.94	(0.85–1.05)	0.284
Low	1.04	(0.95–1.13)	0.396	1.07	(0.98–1.17)	0.108
**Long-term resident**	ref			ref		
**In-migrant**	0.70	(0.63–0.78)	**0.000**	0.75	(0.67–0.83)	**0.000**
**Community type**						
Agrarian	ref			ref		
Fishing	1.02	(0.94–1.12)	0.639	1.08	(0.98–1.18)	0.107
Trading	0.97	(0.88–1.08)	0.626	0.98	(0.88–1.09)	0.700

^a^Adjusted for variables that were statistically significant in the bivariate analysis and those that were potential confounders (age, sex, marital status and migration status). Bold values indicate *p* < 0.05.

Table 4 shows the unadjusted and adjusted PRR of initiating ART. Men were 25% less likely to be on ART compared with women (adjPRR 0.75, 95% CI 0.69–0.82). ART use was lower in HIV-positive participants aged 15–24 than those aged 30–39 (adjPRR 0.69, 95% CI 0.60–0.80). Never married HIV-positive participants were less likely to be on ART than married persons (adjPRR 0.80, 95% CI 0.66–0.95), and ART use was 24% lower among in-migrants than long-term residents (adjPRR 0.76, 95% CI 0.67–0.85). There were no differences in ART initiation in trading communities (adjPRR 1.00, 95% CI 0.89–1.12) or in fishing communities receiving test and treat (adjPRR 1.00, 95% CI 0.96–1.17) compared to agrarian communities.

**Table 4.** Unadjusted and adjusted prevalence risk ratio (PRR) for initiating ART, Rakai, Uganda

	Unadjusted			Adjusted		
Characteristic	PRR	95% CI	*p*-Value	PRR^a^	95% CI	*p*-Value
Female	ref			ref		
Male	0.81	(0.75–0.89)	**0.000**	0.75	(0.69–0.82)	**0.000**
**Age, Years**						
15–24	0.66	(0.58–0.76)	**0.000**	0.69	(0.60–0.80)	**0.000**
25–29	0.76	(0.68–0.85)	**0.000**	0.79	(0.70–0.88)	**0.000**
30–39	ref			ref		
40+	1.04	(0.94–1.15)	0.436	1.05	(0.95–1.16)	0.361
**Marital status**						
Married	ref			ref		
Never married	0.74	(0.62–0.88)	**0.001**	0.80	(0.66–0.95)	**0.014**
Previously married	1.02	(0.93–1.11)	0.645	0.95	(0.87–1.03)	0.230
**Religion**						
Christian	ref			ref		
Muslim	0.99	(0.87–1.13)	0.923	0.99	(0.87–1.12)	0.849
Other	0.95	(0.58–1.47)	0.844	0.98	(0.59–1.51)	0.930
**Occupation**						
Agriculture	ref			ref		
Home/casual/other	1.02	(0.90–1.15)	0.777	1.02	(0.91–1.16)	0.698
Shop/skilled worker	0.90	(0.75–1.07)	0.239	0.97	(0.81–1.16)	0.743
Bar/waitress/sex worker	0.99	(0.85–1.15)	0.889	0.99	(0.85–1.16)	0.921
Fisherman	0.81	(0.70–0.94)	**0.006**	0.97	(0.82–1.15)	0.722
Trade/truck or motorcycle driver	0.95	(0.83–1.09)	0.473	0.99	(0.86–1.13)	0.846
**Wealth Index**						
High	ref			ref		
Middle	0.93	(0.83–1.04)	0.194	0.93	(0.83–1.04)	0.195
Low	1.00	(0.92–1.10)	0.959	1.05	(0.95–1.15)	0.344
**Long-term resident**	ref			ref		
**In-migrant**	0.71	(0.64–0.80)	**0.000**	0.76	(0.67–0.85)	**0.000**
**Community type**						
Agrarian	ref			ref		
Fishing	1.00	(0.90–1.10)	0.941	1.06	(0.96–1.17)	0.255
Trading	1.00	(0.89–1.12)	0.880	1.00	(0.89–1.12)	0.965

^a^Adjusted for variables that were statistically significant in the bivariate analysis and those that were potential confounders (age, sex, marital status, and migration status). Bold values indicate *p* < 0.05.

In this cohort, we found the UNAIDS “90–90–90” treatment targets to be 98-65-92: among people living with HIV in the RCCS 98% (3577/3666) received HCT, 65% (2312/3577) of HIV-positive participants diagnosed had initiated ART, and 92% (336/366) on ART who had a viral load measurement were virally suppressed at 12 months. We found the “‘90–90–90” treatment targets to be 98–69–93 for women and 97–57–89 for men.

## Discussion

Using the HIV care cascade to identify gaps and opportunities for quality improvement is important for programme evaluation. Most HIV care programmes are clinic based and do not have population-based data with which to determine the characteristics of HIV-positive individuals who do or do not access care. In contrast, the RCCS provides information on patient-level factors affecting HIV care utilization. Our results indicate disparities in engagement in HIV care among several sub-populations.

HIV testing and knowledge of HIV status was high as persons in the RCCS are offered immediate HIV results based on a rapid test algorithm. The high uptake of HCT in this setting was likely due to the community-based HIV testing strategy, and ongoing health education which strongly recommends receipt of results. However, underutilization of HIV care services remains a substantial problem in this setting, despite the availability of free services in close geographic proximity. When comparing our findings to a recent analyses of the HIV care cascade in the Rwanda, we found lower rates of enrolment into care (76% vs. 86%), but higher rates of ART initiation among those enrolled (85% vs. 63%), and higher rates of viral suppression among those who were retained in care (92% vs. 82%)[25]. We defined viral suppression as 1000 copies/mL per WHO guidelines, in order to adopt a standard measure and allow comparability across studies. However, sensitivity analyses lowering the viral load to the assay limit of detection (400 copies/mL), did not change our estimates of viral suppression. The majority of persons enrolled in care had initiated ART, and this was similar in communities using a CD4 cell count ≤500 for ART initiation as well as in fishing communities using test and treat. These findings are supported by the recent results of the ANRS 12,249 treatment as prevention trial that found delayed enrolment into care reduced the potential benefit of early ART initiation [26], suggesting that implementing the new 2015 WHO guidelines, recommending universal access to ART regardless of CD4 count, may not be effective in increasing ART coverage. As treatment for all is implemented in Sub-Saharan Africa, programmes will need to focus on enrolment into care that is the most critical area in the cascade.

Consistent with other studies, we found that men had lower engagement in care than women [27–29] and that younger age was associated with lower engagement in care [27,29], which indicate a need to target interventions for these subpopulations. The lower ART initiation among youth could be a function of earlier stage infection, and lower rates of ART eligibility. However, we found the same disparities by age in the fishing communities where ART eligibility is based on test and treat. The finding that new in-migrants underutilized care is corroborated with other studies [30–32]. However, it is unclear whether migrants are care-naïve, or whether their care and treatment was interrupted by their migration. Nevertheless, there is a need for interventions to effectively link new in-migrants with HIV care and treatment. These findings support the need for rigorous implementation science, and qualitative studies to discover the underlying reasons why some subpopulations are at higher risk of not linking to care than others and to better understand barriers of service use within these subgroups.

This study has several limitations. First, we cannot be sure that the cascade estimated from the RCCS population can be generalized to the total HIV-positive population of Rakai District. However, the distribution of behaviours in the RCCS is consistent with rates from the Uganda National HIV Serosurvey and the Uganda Demographic and Health Survey [33–36] and participation rates in this study were comparable to similar community cohorts in Africa [37]. The proportion of participants who sought care at other facilities or failed to report care to avoid stigma is not known. In addition, previous studies have shown conflicting results regarding the accuracy of self-reported utilization of healthcare among HIV-positive individuals [38–41]. However, the use of a combination of data sources is the most effective method for measuring care outcomes [42–44]. Thus, the use of both clinic-based records and self-reported information from RCCS surveys likely reduced measurement error. We used self-reported use of ART, but a previous study of self-reported ART use validated by detection of plasma antiretroviral drugs in this study population found a high specificity (99%) and sensitivity (76%) for self-reported ART use [45]. Missing data on viral loads among patients 12 months after ART initiation was a further limitation. This was due to programmatic delays in the return of results, so we extrapolated available plasma viral load data to the proportion on ART. Nevertheless, inverse probability weighted analyses to adjust for differences between patients with and without viral load results suggested that this extrapolation was unbiased.

## Conclusions

In the four areas of the HIV care cascade we assessed, 98% were aware of their HIV status, however, the remaining three areas were below global targets, likely due to both an ART eligibility criterion of <500 CD4 cells/mL, and suboptimal entry into care for several sub-populations. Interventions are needed to promote enrolment of HIV-positive males, younger individuals, and in-migrants into HIV care which will require new resources and strategies to meet global targets for ART initiation, retention, and viral suppression.

## Notes

To access the supplementary material to this article please see Supplementary Files under Article Tools online.
